# Disrupting Work and Leisure in Lockdown: the Case of the Soho Poly

**DOI:** 10.1007/s41978-023-00135-z

**Published:** 2023-06-03

**Authors:** Matthew Morrison, Guy Osborn

**Affiliations:** University of Westminster, Westminster, UK England

**Keywords:** Lockdown, Culture, Leisure, Time, Space

## Abstract

This article takes as its point of departure the impact of Covid 19 on leisure and work and uses the London arts venue the Soho Poly as a lens through which to explore the profound disruption the pandemic represented. Beginning with a survey of the Soho Poly’s origins in the early 1970s, the authors demonstrate how these laid the groundwork for the venue’s current artistic policy of ‘disrupting the everyday’ with arts and culture. The authors then examine the Soho Poly’s output during 2020 and 2021 and suggest that key philosophies of temporal and spatial disruption in some senses found their moment in the particular circumstances of lockdown. Drawing on this observation, the authors consider how some of the discoveries prompted by the pandemic might be used by arts providers to rethink the ways in which arts and culture can continue to deconstruct, and disrupt, outmoded divisions between work and leisure.

## Disrupting Work and Leisure in Lockdown: the Case of the Soho Poly

The UK’s national lockdown, which began on 23 March 2020, immediately disrupted long-held notions of space and time.^1^ For those confined to their homes, it was suddenly necessary to reconsider when and where expected daily activities were supposed to take place, if indeed they were supposed to take place at all.^2^ Although, for many, working life was abruptly imported into the domestic sphere, for others it was paused entirely with the introduction of the British government’s furlough scheme.^3^ For those with children, the closure of schools meant increased childcare and home learning responsibilities. At the same time, many activities traditionally viewed as leisure – visiting friends, playing sport, going to cinemas and museums – were outlawed (Morris & Orton-Johnson, 2022, 2). As Roberts noted at the time, ‘[i]t takes longer to list activities that were prohibited or inaccessible under lockdown than uses of leisure that remained legal and accessible’ (2020, 618). Under the pressures of lockdown, therefore, established spatial boundaries between settings associated with work, education, childcare and leisure were dissolved, whilst time itself, in Shir-Wise’s evocative phrase, seemed to melt (2022, 221). Such changes created anxiety for those whose professional life encroached on valued leisure time, so often articulated as an ‘antidote’ to work (Mansfield et al., 2020, 2) and also for those for whom a culture of long working hours was associated with status and economic success (Shir-Wise, 2022). Lockdown meant that work and leisure became more ‘intertwined’ than ever (Sivan, 2020, Miah, 2011, Clarke and Crichter, 1985) and, as Adisa et al. demonstrate with reference to a study exploring boundary management amongst academics during the pandemic, the ‘physical and temporal integration’ of ‘segmented work and family domains’ was a source of strain (2022, 1701).

As the country adjusted to lockdown life, however, activities emerged in response to the country’s transformed spatial and temporal circumstances. Morris and Orton-Johnson (2022, 17) have written about the phenomenon of camping at home, which ‘challenged normative understandings of our homes as domestic spaces’. Here, rather than physically travelling in the pursuit of adventure, families imaginatively disrupted the home space itself. King and Dickinson (2023) have also written about the ways in which local and perhaps previously neglected environments (urban parks, playgrounds, common land) became sites for new kinds of ‘meaningful actions’. Meanwhile, virtual spaces were embraced as never before. Platforms including Zoom, Teams and Google Hangouts provided online meeting places, while everything from plays and concerts to exercise workshops and education was accessed through computers, phones and televisions.

In the sphere of the arts and cultural industries, with which this article is concerned, spatial and temporal disruption occurred simultaneously to the working patterns of producers and to the leisure patterns of consumers. As Osborn wrote in 2020:

The lockdown has had a brutal effect on live performance and culture. Even before the formal restrictions were announced, venues and galleries began closing, and it almost immediately became apparent that how we consume culture was to change for the foreseeable future, and perhaps forever.

One such venue was the Soho Poly in London’s Fitzrovia, which operated as an alternative, or ‘fringe’, theatre between 1972 and 1990 and has enjoyed a second life since 2012 under the auspices of the University of Westminster.^4^ The Soho Poly makes for a compelling study in the context of the pandemic and national lockdown because of the ways in which it has consciously sought, through different phases of its history, to activate its own spatial and temporal disruptions. As a tiny basement theatre, originally offering plays at lunchtime, it explicitly confronted assumed notions of when and where culture, considered here as a leisure activity, should be enjoyed. More recently, the practice and policy of the revived Soho Poly, led by creative-producers (and article authors) Dr Matt Morrison and Professor Guy Osborn, demonstrate an approach to cultural production that values the permanent disruption of time and space above attempts to rethink or reimagine where boundaries between work and leisure might lie.

This article begins with an overview of the Soho Poly and its disruptive practices. With the exception of Morrison’s 2017 monograph, little has been written in this area and the venue’s history is therefore outlined in some detail to provide a meaningful context for the ways in which Morrison and Osborn have sought to reimagine its original ethos in their more recent programming. The article then examines the crisis moment of March 2020 and some of the ways in which cultural venues responded. It addresses the creative decisions made by the Soho Poly during lockdown itself, with particular reference to an artistic philosophy rooted in the value of ‘disrupting the everyday’ with arts and culture, and offers ways forward for arts venues in the light of discoveries made during a time of national tumult. Overall, the paper forms part of a practice-as-research investigation which Morrison and Osborn have been engaged in since 2012, and which has been supported at different stages of development by the National Lottery Heritage Fund, Westminster City Council, The University of Westminster and the Being Human Festival. ‘The Soho Poly Project’ therefore operates across disciplines and methodological approaches, offering a narrative account that adds to the ‘scholarly noise’ (Lamond & Lashua, 2021, 10) around the animation of leisured space.

## The Soho Poly, 1972–1990: Disruptive Theatre

In 1968, The Soho Theatre Company was founded by Fred Proud and Verity Bargate. The company produced early work at the Open Space theatre on Tottenham Court Road, the basement of a Chinese restaurant in New Compton Street, and the back room of the King’s Head pub in Islington. In 1972, Proud and Bargate were introduced to another basement, this time on Riding House Street, owned by the then Polytechnic of Central London, now the University of Westminster. Striking a deal with the institution, they moved their company into the space, a former student arts workshop and folk music venue. For the next 18 years, the newly named Soho Poly was to forge a reputation as one of the capital’s most important alternative theatres, nurturing writers, actors and directors including Hanif Kureishi, Simon Callow, Harriet Walter and Barrie Keeffe.^5^ Most notably, the Soho Poly was one of the pioneers of a new mode of ‘lunchtime theatre’. For most of the 1970s, its plays were enjoyed between 1 pm and 2 pm by an audience nibbling sandwiches or sipping from bowls of homemade soup. Despite such innovations, and the calibre of its creative collaborators, the early history of the Soho Poly has not been widely written about. Part of the reason turns on the venue’s contested history as a ‘radical’ space (Morrison, 2017, 26–32).

Radical practice in the arts is often associated with the political, cultural and artistic aims of individuals or small groups. In turn, these are seen to respond to, and be the consequence of, different forms of cultural, political and socio-economic pressure. In the context of the alternative theatre of the 1970s, this kind of analysis sees the roots of radical practice in the global political events of 1968, including the Prague Spring, the Vietnam War, political assassinations, and the student uprisings in Paris (Bull, 1984; Itzin, 1980). Much of the new work that appeared on Britain’s smaller stages during the 1970s had absorbed the energy of such upheavals and the Soho Poly’s programme was no exception. Early seasons drew on the American counterculture (with plays such as Michael Mclure’s *The Pansy*), produced socialist work by dramatists such as Howard Brenton and David Edgar, and showcased more formally experimental pieces from the European avant-garde. And yet the Soho Poly was not itself seen as explicitly ‘radical’, at least in so far as it did not actively promote a particular credo, advocate for an overtly political or social cause, or champion artistic form-breaking as a concomitant objective. Programmes and press releases of the time point instead to a general commitment to ‘new writing’ and ‘the best new plays’.^6^ Even the more obviously experimental seasons were peppered with ‘conventional’ pieces, light comedies and revived classics. Much has been written about the radical theatre of the period (see for example Itzin, 1980, Ansorge, 1975, Craig, 1980). But by virtue of not being radical *enough*, the Soho Poly and theatres like it have fallen out of the theatrical record.^7^ Also lost from the account is a crucial property of these companies and venues. For while they may not have always seemed suitably ‘radical’, they were profoundly ‘disruptive’. Indeed, the Soho Poly was disruptive in at least two ways: it disrupted time and it disrupted space. Both of these disruptions were to have significant implications on theatrical form and content, on audience demographics and behaviour, and on the idea of leisure itself.

By the time Fred Proud and Verity Bargate had moved into the Riding House Street basement, there were already a dozen or so functioning lunchtime spaces in the capital. And, as lunchtime theatre consolidated its position in the cultural ecology, its proponents became more vocal in advocating for the ways in which they were subverting theatre-going norms. By producing plays in the middle of the day they realised that they had the potential to reach a different sort of audience – local workers, those on lower incomes, people who simply enjoyed the more informal idea of watching a play in their civvies, over a pint and a sandwich on their lunch break. As Rosalind Asquith noted in 1980 (147–148), in doing so, the lunchtime theatres demonstrated a new and intentional engagement with the relationship between work and leisure:

The original intentions [of the lunchtime theatres] were numerous – from the blatantly commercial impulse to showcase the work of new writers and performers in circumstances that were relatively painless economically, to the much more radical motive that, by presenting plays at an unusual time of day, one was breaking through one of the paradigm conventions of Western theatre.^8^

Spatially, too, lunchtime theatres were disruptive by virtue of the unconventional environments in which their work was produced. Like the Soho Poly, most venues were small and very rarely displayed the trappings of bourgeois theatre architecture. There were no proscenium arches or royal boxes in the room above the pub or in a basement cellar. Actors and audiences were forced into close proximity, often sharing the same queue for the bathroom or drinks at the end of the show. The informality of a shared space was just one of the ways in which alternative theatres, in general, worked to shake up tradition, helping to break down hierarchical relationships often rooted in class. But it was on the lunchtime stages that this new way of experiencing theatre was most acutely felt. For a midday show there was no need to hire a babysitter or pay for an expensive pre-theatre dinner. And as you watched the drama, starring actors who were within touching distance, you weren’t expected to sit in deathly silence; impossible whilst slurping soup or enjoying a drink. Lunchtime theatre, then, disrupted theatrical conventions around the ‘proper’ time of performance and the length and form of dramatic work. It disrupted the space in which plays were expected to take place, and the ways that audiences were expected to receive them. And it offered the whole package to a different sort of audience, including those for whom evenings were unavailable. Inevitably, that often meant women; Shirley Barrie, a mother of two young children, was just one of those directors who were liberated by the possibilities of the lunchtime slot (Morrison, 2017, 115). Most significantly, the work of the Soho Poly and the other lunchtime theatres presented a profound challenge to the idea that the enjoyment of the arts belonged to a defined ‘leisure time’, marked out in opposition to ‘the working day’.

## The Soho Poly 2012–2020: Overlap, Multiple Valency and Co-creation

Following eviction from Riding House Street by the Polytechnic in 1990, the Soho Theatre Company survived peripatetically by producing plays in venues across London. By the end of the 1990s they had secured National Lottery money to pay for the conversion of a former synagogue on Dean Street to create a purpose-built theatre and bar complex. Soho Theatre remains on this site today. Meanwhile, the original Soho Poly venue in Fitzrovia slipped into disuse, becoming little more than a University storage room. In 2012, however, it was rediscovered by doctoral researcher Matt Morrison in the course of organising a festival marking 40-years since the venue’s foundation. Taking place on site across three days in June, the festival included talks, panel discussions and the presentation of short plays by theatre company The Miniaturists.^9^ But despite the momentum generated by these anniversary celebrations, the basement had been revealed to be in a state of considerable disrepair and for the next five years it remained dark while a campaign for building works was mounted.

During this period, the role of public engagement was becoming more prominent in the Higher Education sector (Watermeyer, 2011, 2015) and, in 2017, a new opportunity arose for the Soho Poly, now partially renovated, to apply for funding from the Being Human Festival (Being Human, undated). This festival, founded in 2014, is an annual celebration of humanities subjects and their impact outside the academy led by the School of Advanced Study at the University of London and sponsored by the British Academy and the Arts and Humanities Research Council. The theme of Being Human 2017, Lost and Found, was well aligned with the processes of physical and archival excavation which had underpinned Morrison’s original research and the funding also facilitated artistic experiments drawing on the disruptive ideas which had defined the venue’s early output.^10^ The Being Human Festival now became a regular strand of work for the re-emerging Soho Poly, helping to shape a programming policy that continued to reference the shifting and ambiguous relationship between work and leisure.

Inspired by their participation in the 2017 festival, for example, Morrison and Osborn began parallel research into the University of Westminster’s musical heritage (Glew et al., 2013; Molden, 2018). Underpinned by the idea that archival imprints linger in the spaces where performances have taken place (see, for example Reason, 2003), they mounted a new series of ‘ghost gigs’. For each, recordings were sourced of concerts that had occurred at various sites associated with the University of Westminster’s history. These recordings were replayed on significant anniversaries in the places where the concerts had been originally performed. The first ghost gig took place, therefore, not in the Soho Poly, but in the refectory of the University of Westminster’s New Cavendish Street campus. This space had previously been a performance venue at the University, playing host, in 1985, to the band New Order. At 1 pm on 6 December 2018, staff at the University were invited back to the space (another basement) to hear the replay. Lunch was provided, and no-one was expected to stand around in reverential silence listening to the scratchy recording coming out of an old cassette deck. Instead, here was an opportunity to mingle and to chat with colleagues, the intention being, in part, to inspire a ‘watercooler moment’ – a revealing phrase that picks out the way in which discussion of (often but not exclusively) cultural events can spill into people’s working lives. The music itself was both focus and background and the question became, was this New Order ‘gig’ a work event or a cultural event? Both? Or somewhere in between?

Other events during this period also exhibited degrees of overlap or multiple valency. For the following year’s Being Human Festival (2018, theme: Origins and Endings), the Soho Poly’s original artistic director Fred Proud was invited to direct a reading of the first play produced by his Soho Theatre Company, Friedrich Durrenmatt’s *One Autumn Evening*, performed in 1968 at the Open Space Theatre. By asking Fred Proud to revisit this work, Morrison and Osborn were consciously engaging him in a practice-as-research experiment designed to activate personal memories. Invited partly to test the properties of lunchtime theatre in one of its original sites, the audience, too, were contributing to a form of historical re-enactment. The production was simultaneously, therefore, a public performance and an embodied component of Morrison and Osborn’s academic research.

Appropriately given its theme (Digging Deeper), Being Human 2019 provided further opportunities for exploring the intersections of work, leisure, research and performance. Events included a lunchtime concert by singer-songwriter Martin Stephenson, an exhibition on the relationship between Pop and Politics, and an afternoon zine-making workshop, at which participants could creatively curate their own memories of personally significant musical events. This last event was noteworthy for the way that it expressed another developing strand of the new Soho Poly’s artistic policy, namely the belief that disruptive events should be rooted in co-creation, further blurring the distinction between work as active production and leisure as passive consumption. Here Morrison and Osborn also owed a debt to the clarion call attributed to the assemblers of Sideburns, a ‘Strangler-zine’, in December 1976. Next to an image of an A chord, an E chord and a G chord was the simple invocation: ‘Now form a band’ (Savage, 1991, 281). The imperative echoes Murdock’s description of radical theatre as a form committed to ‘prompting people to reflect critically on the present situation’, whilst simultaneously ‘encourag[ing] them to take action to change it’ (1980, 152).^11^

The success of these events, evidenced through post-event feedback forms and attendance numbers, now encouraged Morrison and Osborn to attempt something more ambitious: the Soho Poly Arts Club (University of Westminster, 2020). Scheduled to open from March 2020, projected events included film screenings, poetry salons, more ghost gigs, lunchtime play readings, exhibitions and community craft workshops. March 2020 was, however, to prove an inauspicious month to launch such a venture.

## Lockdown Leisure^12^

Cultural institutions, like society itself, respond to trauma in a variety of ways. There is a voluminous literature, for example, on crisis response theory within the context of leisure and tourism (Ritchie, 2009; Ritchie & Jiang, 2019).Whilst the cultural sector was already undergoing rapid change before Covid (Kolvin, 2020; Kolvin & Scholer, 2020), the pandemic accelerated and exacerbated this. Indeed, Kolvin and Scholer (2020, 13) note, echoing the Soho Poly ethos and approach, that leisure venues ‘…need to expand their operations in terms of time and space’.

Considered globally, first responses to the pandemic included maintaining a watching brief, hard lockdown, partial lockdown, border closures, and the stockpiling of food.^13^ In the UK, varying levels of restrictions on movement and public gathering were imposed from 26 March onwards. These emanated largely from the Coronavirus Act 2020 and a series of regulations derived from an urgent power in the Public Health (Control of Diseases) Act 1984 (Barber et al., 2022; Bond et al., 2021; Ormerod, 2020; Samuels, 2020). These regulations included restrictions on movement and gatherings (The Health Protection (Coronavirus, Restrictions) (Steps) (England) Regulations), face coverings (The Health Protection (Coronavirus,Wearing of Face Coverings) (England) Regulations, travel quarantine (The Health Protection (Coronavirus, International |Travel and Operator liability) (England) Regulations) and self isolation (The Health Protection (Coronavirus, Restrictions) (Self isolation) (England) Regulations). Questions were raised at the time about the legitimacy and scope of these (Ormerod, 2020), and whilst the restrictions are now largely revoked or do not apply, the regime as of the end of 2023 is neatly summarised by Liberty (2023).

In addition to possible civil liberty and human rights implications (Liberty, 2023), such restrictions presented acute challenges for the UK’s cultural industries and the impact of Covid on the leisure sector more generally was profound (Sivan, 2020; Banks & O’Connor, 2021; ACE 2020). As Morris and Orton-Johnson (2022, 2) note, ‘nearly all out of home leisure suddenly [became] inaccessible…pubs, nightclubs, cafes… restaurants, heritage sites, galleries, museums, libraries, cinemas, concert halls and theatres, civic and community centres, sports centres and stadiums [closed]’.

Whilst national lockdown provided the first line of defence, the UK’s crisis management went further and deeper. Other government-led responses included the introduction of the furlough scheme, the scheduling of regular press conferences co-fronted by scientific and medical experts, and a marketing campaign to encourage public health and compliance with the new regulations.^14^ With reference to the concept of social flow, Baracsi (2022) makes the interesting suggestion that ‘clap for carers’, initially intended to express gratitude to frontline health care workers, was also co-opted by the government as a form of panic control, bringing people together in shared and affirmative activity. In the early stages of the pandemic, other grassroots-inspired activities arose to counter the effects of social distancing. Necessarily, many of these involved collaboration in the online sphere, but the impulse was also reflected in an upsurge in volunteerism – delivering food, checking on neighbours, fundraising, etc. (Baracsi, 2022). Furthermore, Baracsi and others have argued that lockdown restrictions led to an upsurge in creative thinking within the cultural sector (see also Feder et al., 2022). This can be seen in the sudden proliferation of online cultural events, including the release of previously recorded content, live-streaming of solo performance, online creative workshops and collaborative viewing experiences (Thorpe, 2020). And increasingly, as the pandemic continued, different approaches were combined. A case in point was the Totnes’ Sea Change Festival which, moving online in 2020, presented an eclectic line up of documentaries, live performance and tarot readings, as well as a tie-in with Tim’s Twitter Listening parties. This latter initiative, instigated by former Charlatan’s frontman Tim Burgess, involved a live Twitter feed accompanying the timed playback of classic albums. In a review for The Guardian newspaper, Laura Barton (2020) described the festival as follows, evincing some of the possibilities of online delivery created by lockdown:

The key to the weekend’s success was that by moving across platforms – from video streaming to Soundcloud, Twitter to Spotify to Instagram live – and providing links to explore works further… it managed to create both texture and a sense of companionability. Not once did it feel a flat or lonely endeavour; rather it found a great swell of congregation.

On the day that lockdown was officially announced, Osborn (2020) felt an initial impulse to cancel all Soho Poly Arts Club events. But as the weeks went by, he and Morrison became re-energised by the growing culture of online experimentation, so much of which was having to confront, explicitly, the spatial and temporal expectations around cultural provision. Here their approach followed Lashua et al. (2021) using the pandemic as a form of ‘reversal technique’ to imagine new ideas and opportunities. As a new programme of Soho Poly work began to take shape, it brought with it an opportunity to interrogate how the venue’s own historic challenges to spatio-temporal boundaries might speak to a transformed cultural landscape.^15^

One of the first things Morrison and Osborn returned to was the ghost gig. As previously described, these ‘gigs’ generally took place on an anniversary of the original performances. With reference to the Soho Poly’s history, they were hosted at notional lunchtimes; in practice at a range of ‘unusual’ times throughout the day in recognition of the fact that shift patterns and other atypical work-forms have problematised any standard definition of a ‘lunch hour’.^16^ But if the timing of the concerts was largely unaffected by Covid restrictions, the question of where they were produced was more problematic. In the event, Morrison and Osborn decided to host the concerts virtually by utilising the technology that the University had adopted for the purposes of online teaching – most notably the digital broadcasting site Panopto.^17^ The first two ghost gigs, Ralph McTell and Fleetwood Mac, were streamed ‘live’ in quick succession on 8 and 27 April 2020 respectively. Posters and mini fanzines were produced and made available for download, and listeners were encouraged to live tweet reactions in real time, mimicking elements of the listening party model (Burgess, 2021). In this way, a participatory audience played an integral part in the creation of the event. During later lockdowns the experiment was revived, and in December 2020 following discussion with Burgess’s team, the Soho Poly curated its own Listening Party. Participants were invited to ‘Disrupt their Christmas’ (or, more accurately, to disrupt a working day in the run up to Christmas) with a playlist that drew inspiration from the joint cultural history of the Soho Poly and the University of Westminster.^18^

Whilst it had been possible to transplant ghost gigs into the online world relatively easily, theatrical production was more complex given the need to involve significant numbers of socially isolated individuals. In Summer 2020, however, the Soho Poly mounted Morrison’s intercut monologue play *Dance* as an online production, in collaboration with Brickdust director Charlotte Peters, who was also developing innovative virtual work for Original Theatre Company.^19^ Appropriately, *Dance* was itself a play about online life, but for the new digital version it became necessary to find a form of audience address that would make sense of virtual delivery. With the help of video editor Riaz Gomez, Peters mocked up a YouTube-style video platform, and the two performers (Shonagh Marie and Tim Treloar) spoke as if streaming themselves across social media. The displacement of the production online thereby forced changes to the play which made for a closer alignment between form and content and allowed the story to resonate in new ways.^20^ Also notable was the fact that, if the site of performance was the user’s screen, for the production team, their screens were simultaneously the sites of their work to create it: production meetings, rehearsals and publicity materials were all situated in the same virtual (and visual) field, recalling Clarke and Critcher’s prescient argument that,

…it is the home computer terminal which most clearly represents the potential of the new technology to overturn all our existing ideas of work, leisure and the home. The one piece of technology contains the worlds of work, entertainment, shopping, household responsibilities (accounts), and last, but not least, education. The whole world can be at our fingertips (quoted in Miah, 2011, 138).

In February 2021 the Soho Poly presented another theatrical offering – an online reading of Barrie Keeffe’s ground-breaking play *Sus*, first performed in the physical Soho Poly in 1979. In an after-show discussion, the two interpretations were drawn together across the years thanks to the participation of original director Ann Mitchell and actor Roger Allam; a sense of time travel accompanying the spatial translation of the play to a virtual platform.

Also in 2021, the Soho Poly employed a Research Associate, Dr Helen Eastman, to complete a report on the impact of Covid on arts venues within a mile radius of the Soho Poly (Eastman, 2021). Simultaneously, Morrison and Osborn were making an application for inclusion in the 2021 Being Human Festival (theme: Renewal). Seeing an opportunity to close the research circle, they proposed a creative event that would draw explicitly on Eastman’s work. For this new creative commission – eventually named *Soho After Covid* – Eastman worked with a selection of writers to produce a series of poetic responses to the interviews conducted as part of the Covid report. The poems were then displayed in local shop windows which event attendees were taken to visit as part of a guided tour. Occurring at a time (November 2021) when the majority of the UK population had been vaccinated and most Covid restrictions had been lifted (although before the Omicron surge), this walk proved to be an affirmative and moving participatory experience in which many of the attributes common to the Soho Poly’s recent output – such as overlap, multiple valency and co-creation – were on display.^21^ Here was an event, rooted in research, that also fed back positively into the very issues the research had identified—namely the recent struggle faced by local arts venues and the need to draw audiences back into the West End. The guided walks were spatially embodied, engaging participants with their physical surroundings whilst simultaneously dramatising the way in which lockdown had alienated people from the city. The experience told a story about work, and the difficulties sustaining it during lockdown, in a playful and participatory way. Beginning at 1 pm, the event was itself embedded within working hours (at least as traditionally understood) and offered an invitation to disrupt the day’s normal rhythms and expectations.

## Concluding Remarks: ‘Disrupting the Everyday’ and ‘Everyday Creativity’

As seen above, during lockdown, different areas of our lives including work, leisure, exercise and childcare seemed to blur into one. This undoubtedly caused great friction and stress. But a more positive insight is available too: the realisation that the spatial and temporal boundaries that seem to separate our working and non-working lives have always been susceptible to change, and that this change presents opportunities as well as threats.^22^ Lamond and Lashua (2021, 9) noted that lockdown facilitated and inspired a variety of creative interventions, and inspired ‘…new social rhythms and public connections’. This article has explored such ideas with respect to the Soho Poly and considered what this tells us about the wider relationship between work and leisure.^23^ In lockdown, much more dramatic disruptions to our spatial and temporal conventions have also led to innovation, transforming the ways we experience a multitude of leisure activities and cultural outputs.

Despite this, with the return to something like normality, there have been re-energised attempts to redraw – albeit in a transformed state – clear boundaries between work and leisure; to rethink, in particular, the question of work/life balance. A post-Covid update to a 2019 report from the Henley Business School (based at the University of Reading) found that the pandemic had provided extra impetus to the campaign for a four-day week, with businesses increasingly willing to experiment with implementation. Indeed, in October 2022, one hundred UK companies signed up for a permanent four-day working week for employees with no loss of pay (Kollew, 2022). Although often couched in terms of increased productivity, the UK’s national campaign for such changes also makes explicit reference to wellbeing benefits, including rest and increased leisure.^24^ But whilst such arguments may be compelling, they should not obscure the possibility that the portion of our working lives which is assigned the label ‘work’ – however long or short – might be further enriched by creatively disruptive acts; and that the difficulty in precisely differentiating between work and non-work activities should be positively embraced.

These suggestions align with the conclusions of a 2016 Arts Council England report commissioned from the organisation 64 Million Artists. Revealing that only 8% of the UK population regularly attended funded culture, the report, entitled *Everyday Creativity*, called for a ‘more joined up cultural ecology’ to be achieved through improved support for grassroots initiatives and a more democratic funding infrastructure (Arts Council England, 2016). Crucially, the report acknowledged the fact that ‘[m]any groups talked about the importance of “smuggling” creativity into everyday life (17).’ The report also re-enforced the link between creativity and sociability, noting that: ‘[A]n opportunity to come together with friends, or to meet new people was cited as a core factor in cultivating a culture of everyday creativity’.^25^

Also of critical importance in the *Everyday Creativity* report, is its emphasis on grassroot-led initiatives, not least because alarming post-Covid studies have suggested that the move to online cultural provision did not, as hoped, significantly impact audience reach or effect demographic change. For example, Feder et al., (2022, 2) note that ‘as cultural consumption moved online and to digital modes of delivery and engagement as a result of the pandemic, there was no discernible transformation in the stratification of cultural participation in England’. The philosophy of everyday creativity, however, offers hope for greater engagement by means of a different (co)creative approach, seeking to find ways to encourage small groups and individuals to initiate and manage their own arts and cultural projects. Whilst existing venues continue to struggle to widen the reach of their cultural provision, whether by digital or physical means, here is another way for people to experience arts and other creative leisure pursuits. Indeed, this idea underpins the Soho Poly’s current plans for the first (Westminster City Council-supported) Disrupt your Everyday festival, incorporating a local business creativity challenge whereby local organisations will be encouraged to uncover the secret artists (musicians, writers, painters) amongst their employees, and to give them a spot to perform or share their creative practice in their places of work. In homage once again to the early disruptive ethos of the Soho Poly, ideas about the proper place and time for performance will be overturned as, across one whole day, arts and culture will be brought into the very heart of ‘ordinary’ working life.

## Footnotes

1 The British Prime Minister Boris Johnson announced lockdown on this day, but lockdown measures did not legally come into force until 26 March.

2 Of course, many people – healthcare professionals, public sector workers, etc. – were not in this situation.

3 A subsidy whereby employers could continue to pay staff who were unable to perform their work duties from home. See for example, here, the details in House of Commons Research Briefing (2021) *Coronavirus Job Retention Scheme.*

4 ‘Fringe’ is a contested term. Many small-scale alternative theatres were in fact centrally located geographically (in Central London for example) and arguably culturally too.

5 The name was formed by amalgamating *Soho* Theatre and *Poly*technic.

6 Programmes and press releases in the authors’ private collection.

7 Very little has been written about theatres like the Act Inn, for example.

8 Note, here, that Asquith does make a case for the radical nature of this disruption.

9 A copy of the full programme exists in the authors’ private collection.

10 One of the highlights of the five-day programme was a piece of ‘headphone’ theatre which made use of binaural sound to explicitly interrogate ideas of spatio-temporal dislocation. Audiences were invited to listen to the binaural (sometimes referred to as ‘3D’) recording of a dancer and in so doing to question the reality of the physical space they were occupying. As Hannah Bruce explained in programme notes: ‘You can hear the dancer's presence, but you can also see her absence. We wanted to explore what happens when you inhabit one 'real' space, but with two moments-in-time superimposed virtually upon each other (i.e. the past moment when the dancer was actually there, versus the present moment when you are actually here, and she is only virtually here).’ A full copy of these programme notes exists in the authors’ private collection.

11 For alternative perspectives, see, for example, Hong-An Wu’s related discussion of video game co-creation and prosumerism (2016). Here controversy surrounds the extent to which prosumerism (participants as consumers and producers) can be monetised within neo-liberal economies.

12 This piece focuses on the impact of *Covid* disruption. The point was well made by a helpful anonymous referee that the Soho Poly, in common with other cultural institutions, will have faced many other disruptions historically. These might include responses to economic and financial trauma, labour crises or the shifting tectonic plates of higher education on a macro level. Closer to home, they might include other local ‘disruptions’ such as the impact of gentrification on Soho and Fitzrovia. These lines of inquiry are noted here as worthy of separate in-depth analysis.

13 For a broad survey: https://ourworldindata.org/policy-responses-covidc [last accessed 6 February 2023].

14 Stay Home. Protect the NHS. Save Lives https://www.gov.uk/government/news/new-tv-advert-urges-public-to-stay-at-home-to-protect-the-nhs-and-save-lives

15 This notwithstanding the material threats to its survival that the pandemic had introduced. Lockdown occurred at a critical moment for the campaign to restore the Soho Poly. Specifically, the challenge had become how to convince the University of Westminster of the benefits significant financial investment would provide. Suddenly it was impossible to produce in-person events that would demonstrate the potential of the space.

16 What is lunchtime in London may be breakfast time in Chicago and teatime in Mumbai, a fact that was increasingly relevant as the ghost gigs moved to a streaming model and viewing was, in theory, unconstrained by physical location.

17 For details of this specific platform see https://www.panopto.com/ [last accessed 6 February 2023].

18 See more here, and the event can still be experienced now https://www.lostinmusic.org/replay/index.html [last accessed 6 February 2023].

19 See here https://www.originaltheatre.com/ [last accessed 6 February 2023].

20 The Soho Theatre Company’s early history was full of such examples of dramatic form being positively impacted by the medium of performance. A 1971 production of Joe Orton’s *The Good and Faithful Servant* is a case in point. Originally a television play, director Fred Proud had to find a staging solution to the sheer number of short, televisual scenes. His answer was to create multiple sets, with actors moving briskly between platforms spread around the room. The distinction between actor space and audience space was effectively dissolved, creating an immersive theatrical environment that augmented the intimacy of the storytelling.

21 Event feedback in authors’ private collection.

22 There is an echo here of John’s Dewey contention, expressed in 1916, that the separation of work and leisure, like the separation of playfulness and seriousness, may be a source of psychological harm (see Dewey 1916, as referenced by Breunig, 363).

23 Although the attention here has been on lunchtime work, there were also strands of early-evening and late-night performance during this period.

24 See here the work of 4 day week, the group campaigning for a four day week https://www.4dayweek.co.uk/

25 Walker argues, in an article for The Journal of Positive Psychology, that the proposed value of shared cultural experiences is further bolstered by social flow theory. Evidencing the result of three separate studies, he concludes that: ‘[s]olitary flow, while quite enjoyable, is not as enjoyable as social flow.’ (2020, 9). Kolvin and Scholer take up a similar argument to advocate for the benefits of the ‘social economy’ and the value of enshrining this in legislation (2020). It might also be noted, however, that there is something of a neurotypical frame here. Social interaction many not always align with wellbeing; nor is the disruption of routines, however creatively, always experienced positively.
